# The Combinatorial PP1-Binding Consensus Motif (R/K)x_ (0,1)_V/IxFxx(R/K)x(R/K) Is a New Apoptotic Signature

**DOI:** 10.1371/journal.pone.0009981

**Published:** 2010-04-01

**Authors:** Angélique N. Godet, Julien Guergnon, Virginie Maire, Amélie Croset, Alphonse Garcia

**Affiliations:** Laboratoire E3 Phosphatases, Unité Signalisation Moléculaire et Activation Cellulaire, Institut Pasteur, Paris, France; Health Canada, Canada

## Abstract

**Background:**

Previous studies established that PP1 is a target for Bcl-2 proteins and an important regulator of apoptosis. The two distinct functional PP1 consensus docking motifs, R/Kx_(0,1)_V/IxF and FxxR/KxR/K, involved in PP1 binding and cell death were previously characterized in the BH1 and BH3 domains of some Bcl-2 proteins.

**Principal Findings:**

In this study, we demonstrate that DPT-AIF_1_, a peptide containing the AIF_562–571_ sequence located in a c-terminal domain of AIF, is a new PP1 interacting and cell penetrating molecule. We also showed that DPT-AIF_1_ provoked apoptosis in several human cell lines. Furthermore, DPT-APAF_1_ a bi-partite cell penetrating peptide containing APAF-1_122–131_, a non penetrating sequence from APAF-1 protein, linked to our previously described DPT-sh1 peptide shuttle, is also a PP1-interacting death molecule. Both AIF_562–571_ and APAF-1_122–131_ sequences contain a common R/Kx_(0,1)_V/IxFxxR/KxR/K motif, shared by several proteins involved in control of cell survival pathways. This motif combines the two distinct PP1c consensus docking motifs initially identified in some Bcl-2 proteins. Interestingly DPT-AIF_2_ and DPT-APAF_2_ that carry a F to A mutation within this combinatorial motif, no longer exhibited any PP1c binding or apoptotic effects. Moreover the F to A mutation in DPT-AIF_2_ also suppressed cell penetration.

**Conclusion:**

These results indicate that the combinatorial PP1c docking motif R/Kx_(0,1)_V/IxFxxR/KxR/K, deduced from AIF_562–571_ and APAF-1_122–131_ sequences, is a new PP1c-dependent Apoptotic Signature. This motif is also a new tool for drug design that could be used to characterize potential anti-tumour molecules.

## Introduction

The dynamic process of signal transduction, which involves the concerted action of both protein kinases and protein phosphatases, is a major mechanism in the control of a wide variety of cellular regulations. The PPP family of serine and threonine protein phosphatases, which includes PP1, PP2A, PP2B, and PP4 through to PP7, is responsible for intracellular phospho-serine and phospho-threonine dephosphorylation. The PP1 family of serine/threonine protein phosphatases has been implicated in the regulation of many cellular processes including apoptosis in mammalian cells [1]. PP1 proteins do not exist as free catalytic subunits in the cell but as oligomeric complexes comprising a catalytic structure (PP1c), exerting enzymatic activity, associated with an interacting subunit [2]. The binding of different subunits to a catalytic structure generates a broad variety of holoenzymes.

Apoptosis-inducing factor (AIF) and Apoptotic Protease-Activating Factor 1 (APAF-1) are two major and distinct effectors of apoptosis. AIF is an important mitochondrial flavoprotein involved in the caspase-independent death pathway. Upon cell insults, AIF is released from the mitochondria and translocates to the nucleus [3]. In contrast, and in response to distinct cell death stimuli, APAF-1 controls caspase activation downstream of the mitochondria [4].

To identify potential PP1-interacting proteins, we previously proposed a concept of PP1-signature based on the simultaneous presence of the two distinct PP1c docking motifs, R/Kx_ (0.1)_V/IxF and FxxR/KxR/K [5]. These motifs which are detectable in most PP1-interacting proteins, were initially identified in certain anti-apoptotic members of the Bcl-2 family [6]. In addition, we also more recently described a novel approach for rational drug design, called DPT, which is based on the intracellular delivery of apoptotic PP1/PP2A interacting peptides. Based on these concepts we demonstrated that introduction of the PP1c-interacting FxxR/KxR/K motif, deduced from Bad, which is an apoptotic member of the Bcl-2 family, triggered cell death in HeLa and Jurkat cells [7].

This study is based on the observation that the human AIF_562–571_ sequence contains 10 amino-acid residues which combine the two PP1 consensus docking motifs R/Kx_(0.1)_V/IxF, and FxxR/KxR/K identified in certain Bcl-2 proteins. Furthermore, based on co-precipitation analyses and apoptotic studies, we propose that the R/K-x_(0.1)_V/IxFxxR/KxR/K consensus sequence deduced from AIF_562–571_ sequences is a new PP1-docking motif that is sufficient to provoke cell death in human cell lines. These results also suggest that this combinatorial PP1c docking motif is potentially a molecular tool for drug design.

## Materials and Methods

### Cell culture and reagents

Six distinct transformed adherent cell (HeLa, PC3, HCT116 p53+/+, HCT116 p53−/−, U-2OS, h-TERT RPE-1) were obtained from ATCC. (HFF), Human foreskin fibroblasts also from ATCC was a gift from Dr. MA Buendia (Institut Pasteur). These cells were cultured in Dulbecco's modified Eagle's medium (DMEM, Invitrogen) as exponentially growing subconfluent monolayers in microplates or in 12, 24 or 96-well plates. Non adherent Jurkat lymphoid T cells (clone E6, ATCC) were cultured in RPMI 1640 glutamax medium (Gibco). All cell lines were cultured in medium supplemented with 10% (v/v) fetal calf serum.

### Peptides

High-performance liquid chromatography-purified NH_2_–biotinylated peptides (purchased from Neosystem) were prepared by solid-phase peptide synthesis then dissolved in DMSO and stored at −20°C pending use.

### Antibodies

PP1 antibodies (Monoclonal anti E9) and Anti-mouseIgG-HRP were obtained from Santa Cruz.

### Kits and Reagent

Lab-Tek II Chamber Slide w/Cover, 8 well (Ref 154534, Nalge Nunc International), streptavidin Horse Radish Peroxidase conjugate (Euromedex,), 3,3′-diaminobenzydine tetrahydrochloride (DAB) of DAB substrate Kit for Peroxidase (Vector laboratories), “complete mini EDTA free” protease inhibitor cocktail from Roche, O-Phenylenediamine dihydrochloride (OPD) tablet from Sigma chemical, Annexin-V-FITC from Roche, DiOC_6_(3) from Sigma, Kit DeadEnd™ Fluorometric TUNEL system product (Promega), FluorSave™ Reagent from Calbiochem, Vectashield mounting medium for fluorescence, with DAPI (Vector Laboratories).

### PP1c-binding assays on cellulose-bound APAF peptides

Overlapping dodecapeptides scanning the partial sequence APAF domain encompassing residues 119–141 with putative PP1c docking motif, were prepared by automated spot synthesis (Abimed, Langerfeld, Germany) onto an amino-derived cellulose membrane, as described [8], [9]. Membrane was blocked using SuperBlock (Pierce), incubated with purified PP1c and after several washing steps, incubated with anti-PP1c antibody followed by PO-conjugated secondary antibody. Spots corresponding to positive peptides were visualized using the ECL system.

### Pull down assays to detect interaction of biotinylated peptides with PP1c

Biotinylated peptides were pre-incubated for 2 h at 100 µM (final concentration with lysate) and room temperature with 30 µl of streptavidin-coated immunomagnetic beads (Calbiochem, San Diego CA). During this time, 1×10^6^ HeLa cells at 90% of confluence per test peptide were washed with PBS, trypsinized, harvested with PBS, washed with 1 ml of PBS, and centrifuged at 600 g and 4°C for 10 min. Cell pellets were lysed 10 min on ice with 400 µl of lysis buffer (50 mM Tris pH 7.4, 200 mM NaCl, 10 mM EDTA, 20% Glycerol, 1% Nonidet P-40, 1 mM phenylmethylsulfonyl fluoride (PMSF), 10 mM NaF, 1 mM orthovanadate, and “complete mini EDTA free” protease inhibitor cocktail from Roche). Lysates were clarified at 13,000 g for 10 min at 4°C and after rotation with supernatant at 4°C for 2 h, biotinylated peptides were pulled down with streptavidine magnetic beads and washed twice with 750 µl of lysis buffer. Bound proteins and clarified lysates were analyzed by western blotting using PP1 antibodies.

### Cellular Localization of biotinylated Peptides

A total of 4×10^4^ exponentially proliferating HeLa cells growing in a subconfluent monolayer were seeded per well on a Lab-Tek II Chamber Slide w/Cover in an 8-well configuration (Ref 154534, Nalge Nunc International) and incubated at 37°C, 5% CO_2_. 48 h later, biotinylated peptides were added to the cells at different concentrations and for different periods of time at 37°C, 5% CO_2_. Cells were washed twice in PBS, and fixed with absolute ethanol for 10 min at −20°C before addition of 100 µM of streptavidin HRP conjugate (Cat # 18-152, Euromedex). After 30 min incubation at 37°C, cells were rinsed twice in PBS and incubated with DAB (Vectorshield) for around 10 min. Cells were washed in distilled water and mounted for microscopic examination.

### Quantification of Peptide Internalization

Before incubation, the peptides were preincubated for 1 h with streptavidin HRP conjugate in a 4/1 ratio. Cells at 80% confluence were incubated with different concentrations of peptide in 24-well plates. After 6 h, cells were washed twice in PBS, trypsinized (Trypsin EDTA, Invitrogen) and harvested in 1 ml of PBS and counted. Cells were lysed in 300 µl of 0.1 M Tris pH 7.4, and 0.5% Nonidet P-40 buffer for 10 min on ice. A total of 50 µl of cell lysate was mixed with 50 µl of OPD buffer (51.4 mM Na_2_HPO_4_ and 24.3 mM citric acid) then mixed with 100 µl of OPD solution (one OPD tablet from Sigma (St. Louis MO) in 100 ml of OPD buffer to which 40 µl of 30% hydrogen peroxide was added just before use). After 10 to 20 min, the reaction was stopped by adding 100 µl of 1 M HCl, and optical density was read at 490 nm.

### Cytotoxicity assays

A total of 3,000 cells were incubated for 24 hours with different peptide concentrations. Peptide cytotoxicity in adherent cell lines was analyzed by a colorimetric assay using 3-(4,5-dimethylthiazol-2-yl)-2, 5-diphenyltetrazolium bromide (called MTT) for adherent cells as described by the manufacturer (Sigma).

### TUNEL Assay

TUNEL assays were performed using the DeadEndTM Fluorometric TUNEL system product (G3250 Promega). A total of 4×10^4^ exponentially proliferating HeLa cells growing as a sub confluent monolayer were seeded per well on a Lab-Tek II Chamber Slide w/Cover in an 8–well configuration (Ref 154534, Nalge Nunc International) and incubated at 37°C, 5% CO_2_. 18 h later, biotinylated peptides were added to the cells at different concentrations and for different periods of time at 37°C, 5% CO_2_. After incubation, cells were rinsed twice with 1X PBS and were fixed with 4% paraformaldehyde solution in 1X PBS for 25 min at 4°C. Slides were rinsed twice with 1X PBS for 5 min and permeabilized with a solution consisting of 0.2% Triton X-100 in 1X PBS for 5 min at room temperature. Cells were washed twice with 1X PBS prior to labeling. Cells are equilibrated with kit “Equilibration Buffer” for 10 min at RT. TUNEL reaction labeling was performed by using the specified amounts of the “Equilibration Buffer”, “Nucleotide Mix” and “rTdT Enzyme” and incubated for 60 min in the dark at 37°C in a humidified chamber. To stop the reaction, the slides were immersed for 15 min in 2X SSC (in 1X PBS) purchased from Promega. Slides were rinsed three times in 1X PBS and mounted with Vectashield containing DAPI. The slides were examined by fluorescence microscopy.

### Annexin-V assays and evaluation of mitochondrial membrane potential (ΔΨm)

Jurkat cells (2.4.10^5^ cells in 400 µl of complete RPMI-1640 medium) were incubated at 37°C and 5% CO_2_ with different peptide concentrations. After 3 h, cells were washed in PBS and we used an Annexin-V-FITC (Roche) to assess outer leaflet phosphatidylserine (PS) exposure in the plasma membrane as a means for the early detection of apoptotic cells. Staining was performed according to the manufacturer's instructions. We also used the cationic Dye DiOC_6_(3), which localizes into intact mitochondria, to measure ΔΨm. DiOC_6_(3) was added at 20 nM for 15 to 30 min at 37°C in the dark. Cells were washed in PBS and immediately analyzed by flow cytometry. A total of 20,000 cells were analyzed in a FACScalibur flow cytometer (BD Biosciences, San Jose, CA).

## Results

### AIF_562-571_, a short c-terminal sequence of human AIF, is a new PP1-interacting sequence

**Figure 1:**
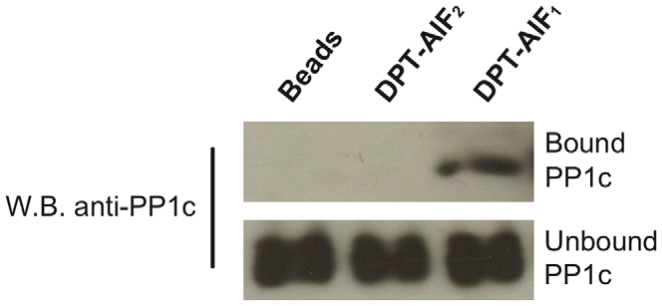
Co-precipitation of PP1c with *DPT-AIF peptides* containing a wild type or single F565/A mutation. PP1c from HeLa cell extracts associated with biotinylated DPT-peptides containing AIF_562–571_ sequences (“bound” panel) in pull-down experiments was identified by immunoblotting with anti-PP1c antibodies. PP1c identification in lysates (“unbound” panel) is shown as a control.

As shown in table 1, the highly conserved c-terminal domain of AIF proteins contains a putative PP1c docking motif, R/Kx_(0.1)_V/IxFxxR/KxR/K, that combines the two previously reported canonical PP1c docking motifs required for PP1c binding in Bcl-x_L_ and Bcl-w proteins [6]. To test the binding capacity of this motif to PP1c, we chemically synthesized DPT-AIF_1_ and DPT-AIF_2_, two biotinylated peptides that respectively correspond to the wild type and to the F566A mutation of human AIF_562–571_ sequence (see Table 2 for details). As shown in figure 1, pull down assays indicated that DPT-AIF_1_ co-precipitates with PP1c from HeLa cell extracts. In contrast, PP1c is not detected in pull down experiments with control beads or mutant peptide DPT-AIF_2_. Similar results are also found with Jurkat cells (data not shown). These data demonstrate that the single exchange of the F566A residue in the R/Kx_(0.1)_V/IxFxxR/KxR/K motif of the AIF_562–571_ sequence is sufficient to specifically prevent PP1c binding.

**Table 1:** Homology of the PP1c consensus binding motifs deduced from the AIF sequence in different species.

SPECIES and PP1 motifs	SEQUENCE OF AIF PROTEINS
Human	_562–_KgViFylRdK_–571_
Mouse	_561–_KgViFylRdK_–570_
Rat	_561–_KgViFylRdK_–570_
Chicken	_540–_KgViFylRdK_–550_
First PP1c consensus docking motif characterized in Bcl-x_L_ and Bcl–w proteins.	(R/K)x_(0,1)_V/IxF
Second PP1c consensus docking motif characterized in Bcl-x_L_ and Bcl–w proteins.	Fxx(R/K)x(R/K)
A new combinatorial Potential motif	(R/K)x_(0,1)_V/IxFxx(R/K)x(R/K)

AIF proteins contain a putative PP1c docking sequence (R/K)x_ (0,1)_V/IxFxx(R/K)x(R/K) which combines the two canonical PP1c docking motifs (R/K)x_(0,1)_V/IxF and Fxx(R/K)x(R/K) initially characterized in certain Bcl-2 proteins [1], [6].

*Sequences are shown with a single letter code for amino acids, and residues corresponding to invariable amino acids in the PP1c site are in capital letters.*

**Table 2:** Origin and sequences of peptides containing human AIF_562–571_.

ORIGIN	ACRONYM	SEQUENCES
AIF_562–571_	DPT-AIF_1_	KgViFylRdK
AIF_562–571_	DPT-AIF_2_ *	KgViAylRdK

A single letter amino acids code is used for all peptides, and residues corresponding to invariable amino acids in PP1c consensus binding sites are in capital letters. *This peptide contains a single F/A mutation that destroys the combinatorial PP1c docking motif.

### AIF_562–571_ is a new cell penetrating and cell death domain

**Figure 2:**
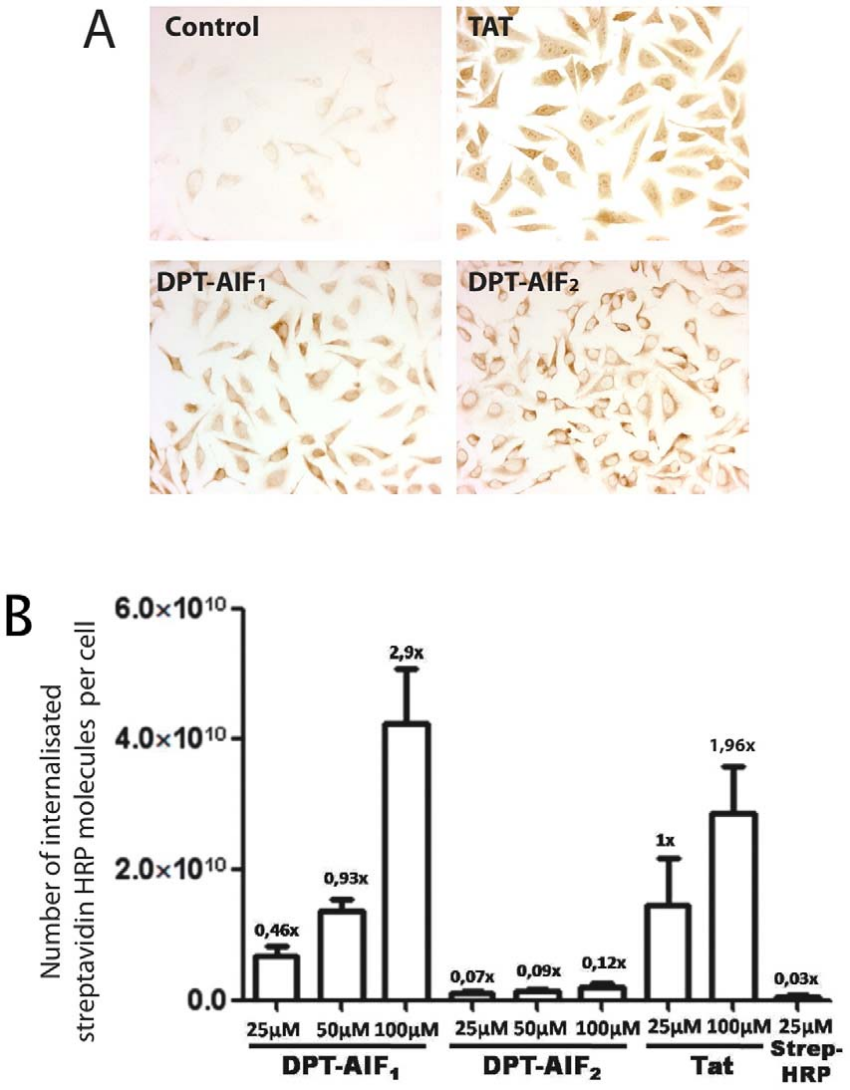
Effect of DPT-AIF peptides on the cell penetration and intracellular delivery of *streptavidin-peroxidase*. (A) In the penetration and localization tests, HeLa cells were incubated with 150 µM DPT-AIF peptides for 2 hrs at 37°C, and before any microscopy, the presence of DPT-AIF peptides was revealed by a colorimetric test DAB. (B) Intracellular delivery of streptavidin-peroxidase by biotinylated- DPT-AIF and TAT peptides in HeLa cells. Streptavidin-peroxidase coupled with biotinylated peptides incubated for 6 hrs at 37°C with internalized complexes visualized by a colorimetric test OPD as indicated in Methods. Statistical analysis was by ANOVA test and significance was set at P<0.05.

**Figure 3:**
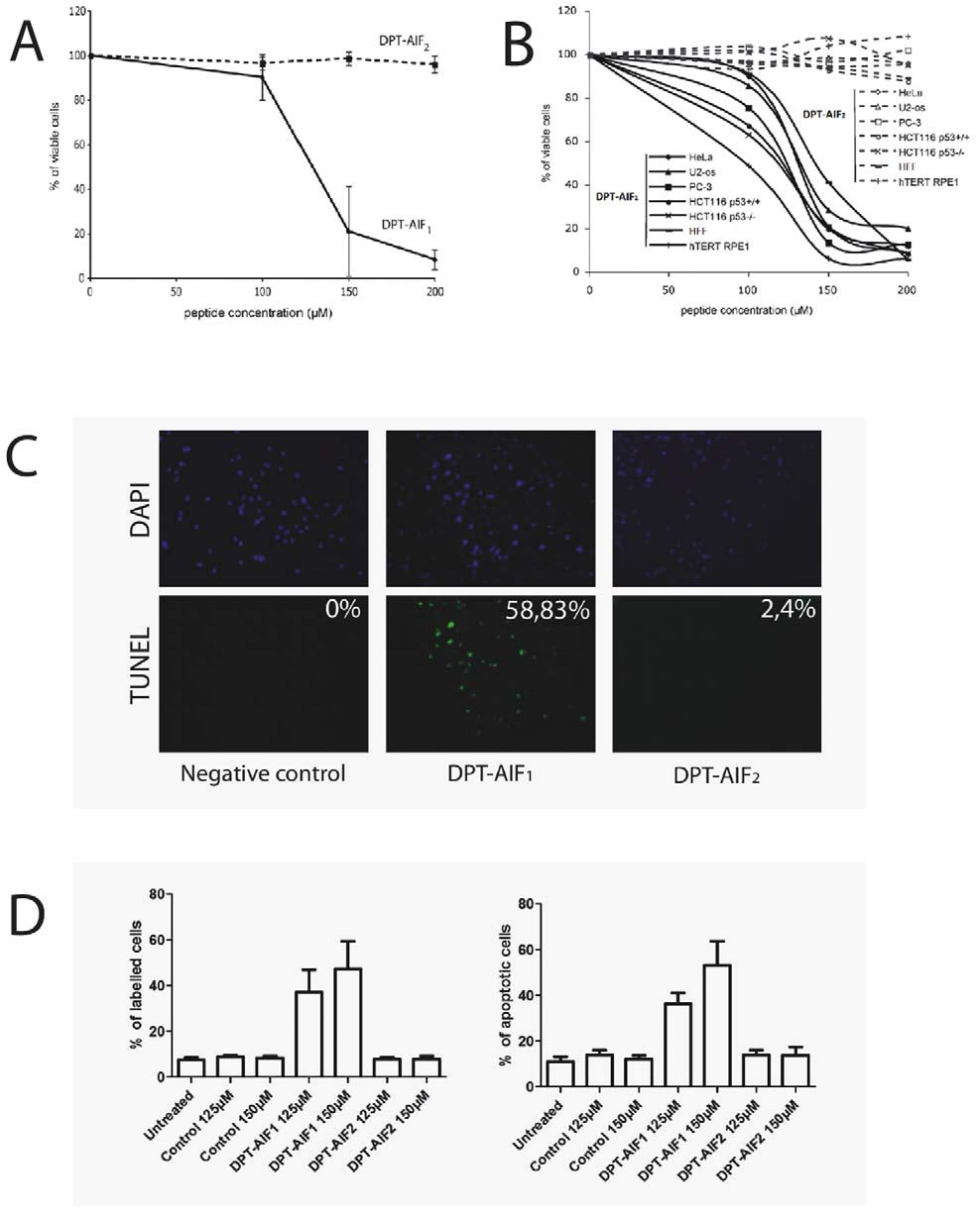
Effect of DPT-AIF peptides on cell survival and cell death. Cell viability assay: (A) HeLa cells, or (B) other adherent cell lines, were incubated for 24 hours with DPT-AIF peptides prior to processing for MTT staining. In A, SD is shown for n = 3, in B for simplicity the figure corresponds to a representative experiment repeated three times. Cell death assay: (C) HeLa cells were incubated for 2 hrs with DPT-AIF peptides prior to processing for the detection of TUNEL positive cells. The % of positive cells was obtained by ANOVA test and significance was set at P<0.05. (D) To monitor apoptosis in Jurkat cells treated with DPT-AIF peptides, we used Annexin V (left panel) or DiOC_6_ (right panel) assays as described in Methods. Statistical analysis was by ANOVA and significance was set at P<0.05.

We previously reported that intracellular delivery of a functional FxxR/KxR/K PP1c-interacting motif, deduced from the sequence of the pro-apoptotic Bad protein, triggered cell death in human HeLa and Jurkat cells [7]. Given that AIF_562–571_ shares a similar FxxR/KxR/K PP1c docking motif, we hypothesized that it might constitute a cell penetrating and cell death sequence. To test this hypothesis, an investigation was conducted into the ability of biotinylated DPT-AIF_1_ and DPT-AIF_2_ peptides to penetrate and deliver a protein marker (Streptavidin-HRP) into HeLa cells. As shown in figure 2A, both DPT-AIF_1_ and DPT-AIF_2_ were localized into the cytoplasm of HeLa cells. However only DPT-AIF_1_, not DPT-AIF_2_, was able to internalize Streptavidin-HRP conjugated molecules even more efficiently than the well characterized positive control TAT peptide (Figure 2B). We also found that DPT-AIF_1_, but not mutant DPT-AIF_2_, was able to induce loss of viability in adherent HeLa cells (Fig. 3A). Similar effects were observed in six other adherent cells (Fig. 3B). More importantly, TUNEL assay showed that DPT-AIF_1_, but not DPT-AIF_2_, induced apoptosis in adherent HeLa cells (Fig. 3C). Experiments using Annexin V or DiOC assays indicated that DPT-AIF_1_, but not DPT-AIF_2_, induced apoptosis in non-adherent Jurkat cells.

Together, these results demonstrate that AIF_562–571_ contains a functional R/Kx_ (0,1)_V/IxFxxR/KxR/K motif that possesses cell penetrating and cell death properties.

### The _564–_VIFYLRDK_–571_ sequence of AIF containing the FxxR/KxR/K motif is sufficient for PP1c binding

**Figure 4:**
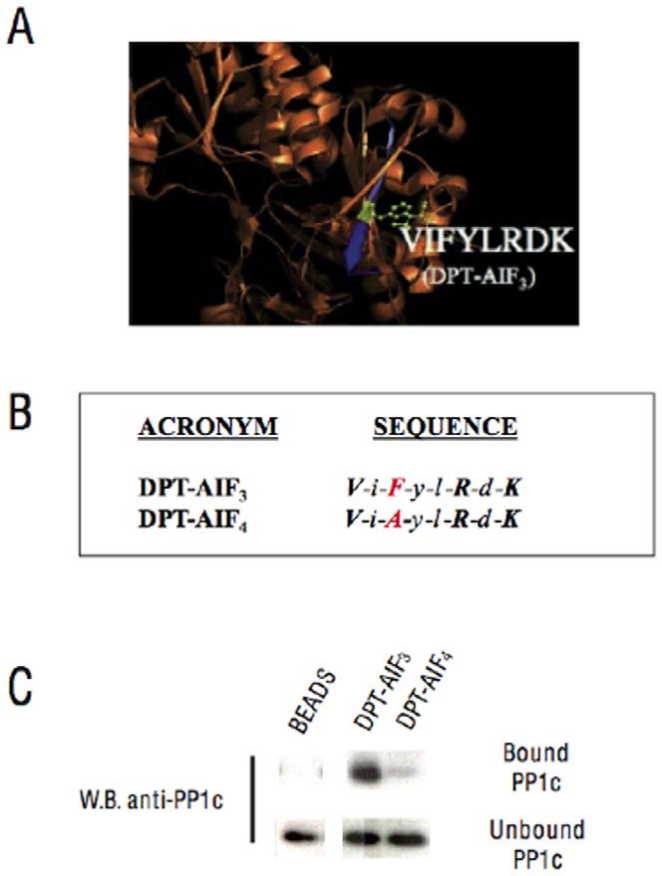
Structural model and biochemical analyses of PP1c interacting sequences in DPT-AIF deletion peptides. (A) Localization of the PP1c binding site in a ribbon representation deduced from the AIF crystal structure obtained from PDB. The PP1c binding site (residues 562–571 in blue) is located in an anti-parallel β-strand. The critical F566 residue mutated in the DPT-AIF_2_ sequence is shown in yellow. (B) DPT-AIF_3_ sequence and DPT-AIF_4_ peptides containing 8 accessible residues of AIF*_564–571_.* (C) Pull down experiments to analyze co-precipitation of DPT-AIF_3_ and DPT-AIF_4_ peptides with PP1c for HeLa cell extracts.

As shown in Fig. 4A, a structural analysis of AIF based on its published crystal structure [10] suggests that the sequence corresponding to residues 562–571 in human AIF adopts a β strand structure. And only the eight residues 564–571 are found to be exposed, suggesting that the minimal _564–_VIFYLRDK**_–571_** sequence may be sufficient for PP1c interaction. To test this hypothesis, we chemically synthesized two distinct wild type and mutated DPT-AIF peptides sharing sequential N-terminal deletion of the human AIF_562–571_ sequence (see Fig. 4B for sequence details). As shown in Fig. 4C, pull down experiments showed that DPT-AIF_3_, which corresponds to the _564–_VIFYLRDK_–571_ sequence containing the FxxR/KxR/K motif, can efficiently co-precipitate PP1c. In contrast, DPT-AIF_4_ containing the F566A mutation in the DPT-AIF-2 sequence was unable to co-precipitate PP1c.

#### Human APAF-1_122–131_ contains a combinatorial R/Kx_(0,1)_V/IxxR/KxR/K PP1c-interacting motif

**Figure 5:**
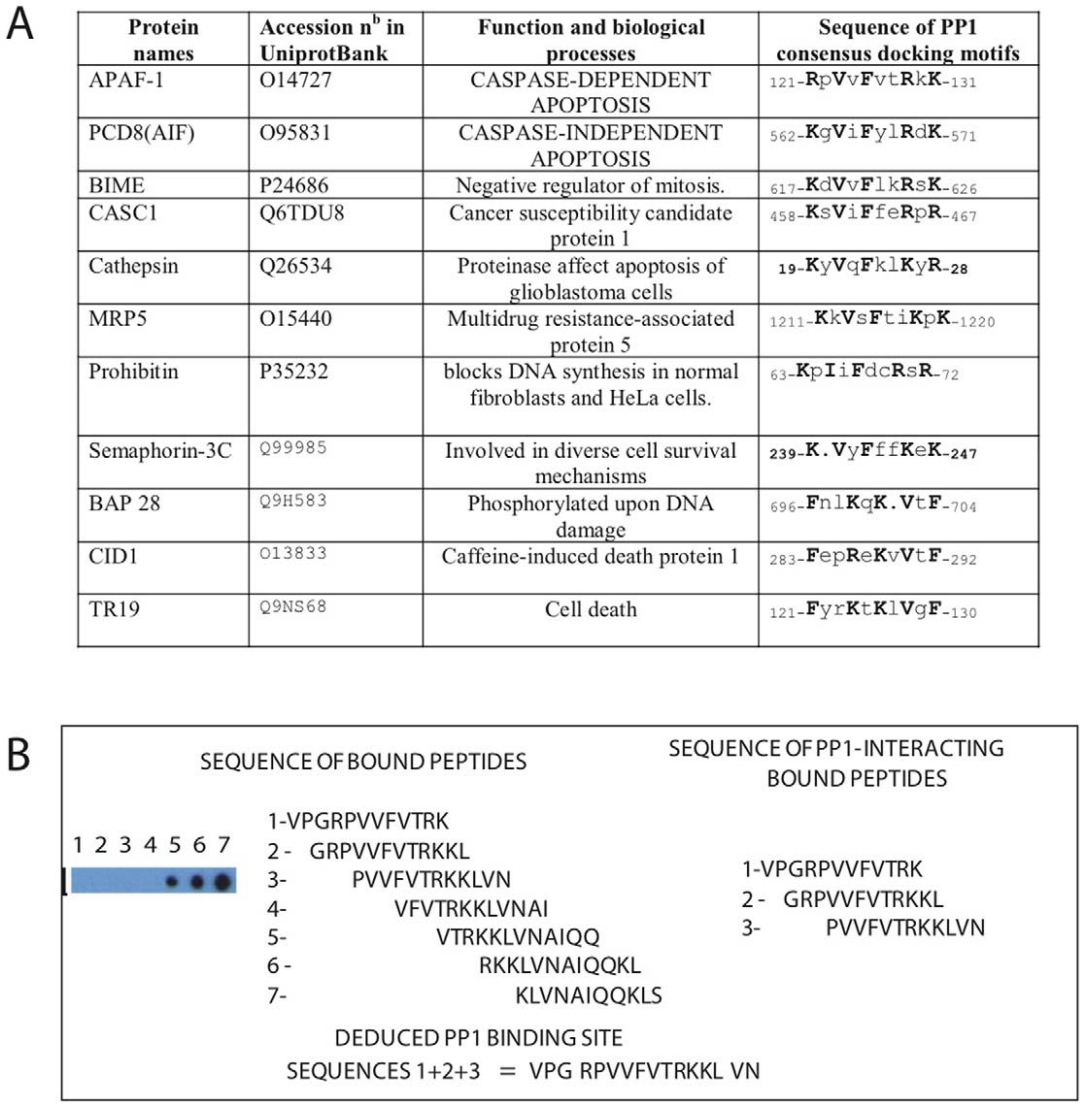
In vitro molecular analysis of the PP1c docking motif in the APAF-1_122–131_ domain. (A) Proteins involved in the control of cell survival pathways with a putative apoptotic signature that combines the two PP1c docking motifs (R/K)-x_(0,1)_-V/I-x-F-and F-x-x-R/K-x-R/K. (B) Autoradiogram of the PP1 binding assay on cellulose-bound APAF-1 peptides. 7 overlapping dodecapeptides with a two-amino acid shift scanning the sequence encompassing aa residues 119–141 of the APAF-1 sequence were synthesized on a cellulose membrane. The membrane was incubated with PP1c holoenzyme and subsequently with anti-1c antibodies followed by peroxidase-labelled anti-mouse antibodies. The residues corresponding to the PP1c consensus binding motif are shown in bold.

As illustrated in figure 5A, the combinatorial PP1c docking motif deduced from AIF_562–571_ is found in the sequence of several proteins involved in the control of cell survival pathways. We therefore hypothesized that APAF-1_122–131_, an APAF-1 domain containing the combinatorial PP1c docking motif deduced from AIF-1_562–571_, can also bind PP1c. Consistent with this hypothesis, a PP1c binding assay showed that APAF-1_122–131_ can bind to PP1c *in vitro* (Fig. 5B). However, in contrast to AIF_562–571_, APAF-1_122–131_ is not a cell penetrating sequence (data not shown).

## Discussion

### AIF_562–571_: a new PP1c interacting sequence with cell penetrating and apoptotic properties

This work is based on our initial observation which suggested that AIF_562–571_, the sequence located in the c-terminal portion of AIF can bind to PP1c trough a potential PP1c consensus docking motif defined by the 10 aa residues R/Kx_(0.1)_V/IxFxxR/KxR/K. Remarkably, this sequence combines two distinct PP1c docking motifs: 1) R/Kx_(0,1)_V/IxF, a well characterized universal conventional consensus motif, and 2) FxxR/KxR/K, a motif that we initially identified in Bcl-x_L_, Bcl-w and Bad proteins [6], [7]. Interestingly, this second motif is also present in most known PP1c binding proteins including inhibitor-2 [5], [11].

It is well established that, in both motifs, the mutation of their unique F residue, usually the F to A mutation, inhibits PP1c binding. Consequently, pull down experiments with DPT-AIF_2_ and DPT-AIF_3_ peptides, respectively containing the F566A mutation and the KV562-563 deletion in the AIF_562–571_ sequence, indicate that the _564–_VIFYLRDK_–571_ sequence containing the FxxR/KxR/K motif is critically involved in PP1c-binding. The PP1c binding site in the R/Kx_(0.1)_V/IxF motif has previously been characterized. Structural studies based on the co-crystal analysis of PP1c and a synthetic peptide containing a synthetic fragment of the muscle GM protein indicated that this motif binds to an hydrophobic channel in the c-terminal region of PP1c [12], [13]. In contrast, the PP1c binding site in the FxxR/KxR/K motif is still unknown. Since both types of motif contain an F and an R/K residue, it is unclear whether the two motifs bind to the same or to a different site on PP1c. Future work based on co-crystal structure studies with a synthetic peptide, containing the _564–_VI**F**YL**R**D**K**_–571_ sequence and PP1c, will be required to determine the binding sequence of a **F**xx**R/K**x**R/K** motif on PP1c. Furthermore, mutagenesis and functional studies of PP1c mutated within the binding sequence of this motif will be used to determine the functional role of the **F**xx**R/K**x**R/K**-PP1c interaction on PP1 activity and on AIF mediated apoptosis.

Surprisingly, AIF_562–571_ also behaves as a new cell penetrating sequence able to internalize HRP complex and induce apoptosis in cultured cells. In addition, similarly to the regulation of PP1c binding, the F566A mutation in DPT-AIF_2_ counteracts these effects. A similar situation has previously been reported with a Bad interacting FxxR/KxR/K motif coupled to inactive non transducing shuttle DPT-sh1 [7]. Together, these data suggest that intracellular transduction is favored by the specific interaction with PP1c. In addition, we confirm that PP1c targeting by a DPT-interacting molecule containing PP1c-interacting FxxR/KxR/K motif is sufficient to induce cell death.

### R/K-x_(0,1)_V/IxFxxR/KxR/K: a PP1-dependent apoptotic signature and new tool for drug design?

Interestingly, and similarly to AIF_562–571_, APAF-1_122–131_, a sequence located downstream of the CARD domain of human APAF-1 proteins, contains a PP1c consensus docking motif defined by the 10 aa residues R/Kx_(0,1)_V/IxFxxR/KxR/K. APAF-1_122–131_ binds *in vitro* to PP1c. However, although it contains a motif similar to AIF_562–571_, APAF-1_122–131_ is not a cell penetrating sequence. The difference between AIF_562–571_ and APAF-1_122–131_ occurs in the non conserved residues within their PP1c docking motif since P123, V127 and D130 in APAF-1_122–131_ are respectively replaced by G, Y and D in AIF_562–571_.

The authors of previous studies have already proposed that short bioactive peptides containing phenylalanine, leucine, and lysine residues could be used in antibacterial, antifungal, and anticancer applications [14], [15], [16]. Other reports have documented anti-tumor effects by cationic synthetic peptides. For example, Araya *et al.*, [17] characterized a 13-mer peptide containing the KKYKAY**F**KL**K**C**K**K polycationic sequence derived from snake venom that exerts anti-tumor effects. Interestingly, this peptide contains a potential **F**xx**R**/**K**x**R**/**K** PP1-docking motif suggesting that binding of this peptide to PP1c may be involved in the apoptotic process.

In this context, the PP1-dependent apoptotic signature motif deduced from the AIF_562–571_ sequence obviously represents a new potential target for drug design. It is noteworthy, as illustrated in Fig. 5A, that this new apoptotic motif is found in several proteins involved in the control of cell survival pathways. Further work will be required to determine the role of this PP1c-dependent apoptotic signature in pathways controlled by these proteins, including AIF and APAF-1 themselves.

In conclusion, our results support the concept of Drug Phosphatase Technology as an original approach targeting PP1/PP2A phosphatases to generate new specific and non toxic cancer drugs. In this study our results established that the PP1 docking motif R/Kx_(0,1)_V/IxFxxR/KxR/K deduced from the AIF_562–571_ sequence represents a potential PP1-dependent combinatorial death motif. We have already characterized DPT-AIF_1_ as a new cell penetrating pro-apoptotic peptide. Future work will be necessary to determine the potential anti-tumor activity of this molecule.
